# CD73-Adenosinergic Axis Mediates the Protective Effect of Extracellular Vesicles Derived from Mesenchymal Stromal Cells on Ischemic Renal Damage in a Rat Model of Donation after Circulatory Death

**DOI:** 10.3390/ijms231810681

**Published:** 2022-09-14

**Authors:** Maria Antonietta Grignano, Stefania Bruno, Simona Viglio, Maria Antonietta Avanzini, Marta Tapparo, Marina Ramus, Stefania Croce, Chiara Valsecchi, Eleonora Francesca Pattonieri, Gabriele Ceccarelli, Federica Manzoni, Annalia Asti, Carmelo Libetta, Vincenzo Sepe, Paolo Iadarola, Marilena Gregorini, Teresa Rampino

**Affiliations:** 1 Unit of Nephrology, Dialysis and Transplantation, Fondazione IRCCS Policlinico San Matteo, 27100 Pavia, Italy; 2 Department of Medical Sciences and Molecular Biotechnology Center, University of Torino, 10126 Torino, Italy; 3 Department of Molecular Medicine, Biochemistry Unit, University of Pavia, 27100 Pavia, Italy; 4 Pediatric Hematology Oncology Cell Factory, Fondazione IRCCS Policlinico San Matteo, 27100 Pavia, Italy; 5 Department of General Surgery, Fondazione IRCCS Policlinico San Matteo, 27100 Pavia, Italy; 6 Human Anatomy Unit, Department of Public Health, Experimental and Forensic Medicine, University of Pavia, 27100 Pavia, Italy; 7 Clinical Epidemiology and Biometry Unit, Fondazione IRCCS Policlinico San Matteo, 27100 Pavia, Italy; 8 Epidemiological Observatory Unit, Health Protection Agency, 27100 Pavia, Italy; 9 Department of Internal Medicine and Therapeutics, University of Pavia, 27100 Pavia, Italy; 10 Department of Biology and Biotechnologies “L. Spallanzani”, University of Pavia, 27100 Pavia, Italy

**Keywords:** kidney transplantation, extracellular vesicles, ischemia-reperfusion injury, mesenchymal stromal cells, CD73, adenosine

## Abstract

We propose a new organ-conditioning strategy based on mesenchymal stromal cell (MSCs)/extracellular vesicle (EVs) delivery during hypothermic perfusion. MSCs/EVs marker CD73 is present on renal proximal tubular cells, and it protects against renal ischemia-reperfusion injury by converting adenosine monophosphate into adenosine (ADO). In this study, after checking if CD73-silenced EVs (EVsi) would impact in vitro tubular-cell proliferation, we perfused kidneys of a rat model of donation after circulatory death, with Belzer solution (BS) alone, BS supplemented with MSCs, EVs, or EVsi. The ADO and ATP levels were measured in the effluents and tissues. Global renal ischemic damage score (GRS), and tubular cell proliferation index (IPT) were evaluated in the tissue. EVsi did not induce cell proliferation in vitro. Ex vivo kidneys perfused with BS or BS + EVsi showed the worst GRS and higher effluent ADO levels than the MSC- and EV-perfused kidneys. In the EV-perfused kidneys, the tissue and effluent ATP levels and IPT were the highest, but not if CD73 was silenced. Tissue ATP content was positively correlated with tissue ADO content and negatively correlated with effluent ADO level in all groups. In conclusion, kidney conditioning with EVs protects against ischemic damage by activating the CD73/ADO system.

## 1. Introduction

The regenerative, anti-inflammatory, and immunomodulatory effects of mesenchymal stromal cells (MSCs) and extracellular vesicles (EVs) derived from MSCs are well known, though the molecular mechanisms underlying these effects are not yet fully understood [1,2,3,4,5,6,7,8,9,10,11]. We previously demonstrated that both MSCs and EVs protect the kidney from ischemic injury by preserving the enzymatic system essential for cellular homeostasis and energy metabolism [12]. MSCs and EVs are known to express high levels of the ecto-5′-nucleotidase CD73, a surface enzyme responsible for adenosine (ADO) production [13]. Moreover, some studies have shown that the immunomodulatory properties of MSCs may be mediated, at least in part, via adenosinergic signaling [14,15].

Under physiological conditions, extracellular ADO levels are low, and mainly derived from the enzymatic phosphohydrolysis of extracellular ATP and ADP [16,17]. During hypoxia, apoptotic or necrotic cells release adenosine triphosphate (ATP) and adenosine diphosphate (ADP), which are converted to adenosine monophosphate (AMP) by CD39 (ecto-nucleoside-triphosphate diphosphohydrolase-1, also known as ectopyrase), which is highly expressed in renal tissues [18]. Subsequently, AMP is converted to ADO by CD73, which is ubiquitously expressed in the kidney, in particular, in the deep cortex and outer medulla region, where the most severe renal ischemia-reperfusion injury (IRI) occurs [19]. Hypoxic conditions increase the expression of CD39, CD73, and ADO receptors [20,21].

ADO is an essential nucleoside for energy production in all cells and tissues. It exerts proliferative and anti-apoptotic effects on renal proximal tubular cells via A1 receptors and on endothelial cells via A2 receptors [22]. ADO acts on renal cells both directly and by replenishing the cellular reserves of ATP [22,23,24,25,26,27]. Recent evidence has shown that the CD73-mediated production of ADO plays a protective role against IRI [28,29,30,31,32,33,34,35]. However, to our knowledge, no study has investigated whether the protective effects of MSCs or EVs in IRI are mediated by adenosinergic signaling. We hypothesized that during renal perfusion with MSCs or EVs, the availability of CD73 increases drastically, generating ADO from the ATP released by inflammatory, necrotic, and apoptotic cells.

We have previously demonstrated that during kidney conditioning in a model of human marginal kidney donation, EVs arrested IRI damage, prevented reoxygenation-dependent injury, and preserved mitochondrial structure and tissue integrity [36]. The identification of all mediators contributing to the best effectiveness of EV delivery during hypothermic perfusion is an important step to improve organ-preservation strategies and transplantation outcomes. To this purpose, in the present study in vitro experiments and a rat model of donation after circulatory death (DCD) were used to determine whether the CD73-adenosinergic axis plays a role in the protective effects of MSCs/EVs against renal ischemia injury.

## 2. Results

### 2.1. MSCs and EVs

**Figure 1:**
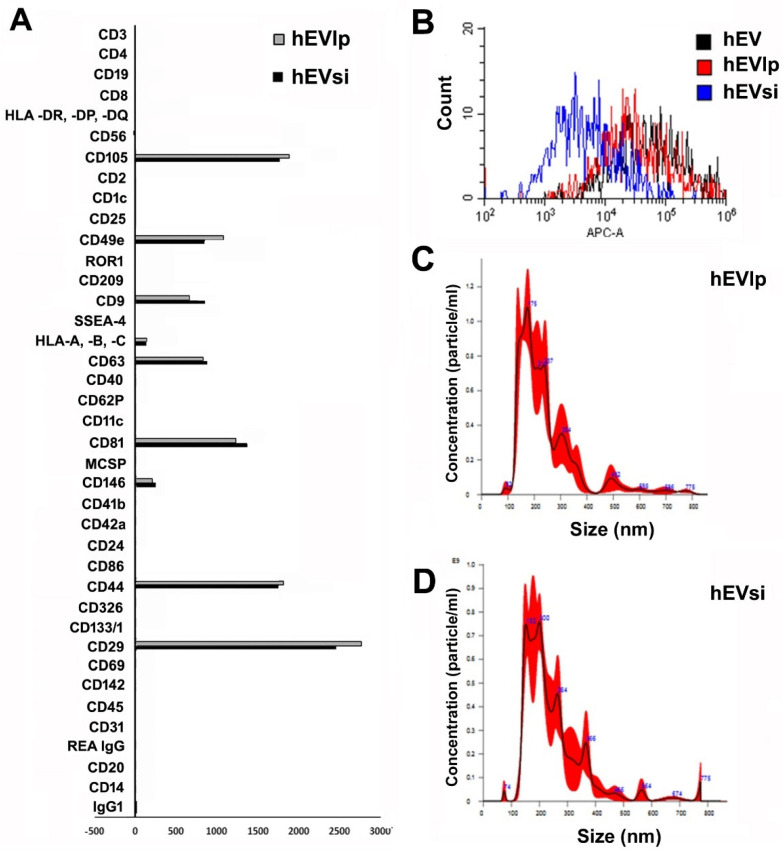
**Characterization of extracellular vesicles (EVs).** (**A**) The surface molecular profiles of hEVlp and hEVsi were determined using a multiplex bead-based flow cytometry assay with 39 multiplexed populations of dye-labeled antibody-coated capture beads. The graph shows the quantification of the median allophycocyanin (APC) fluorescence values for all bead populations after background correction (medium control values subtracted from measured EV values) of representative hEVlp and hEVsi samples (n = 3 samples analyzed with similar results). No differences were observed among the EVs obtained from human mesenchymal stromal cells (hMSCs) transfected in the presence (hEVsi) or absence (hEVlp) of a CD73-specific small interfering RNA (siRNA). (**B**) Representative cytofluorimetric analyses of EVs obtained from naïve hMSCs (hEVs) and from hMSCs transfected in the presence (hEVsi) or absence (hEVlp) of the specific siRNA (n = 3 samples analyzed with similar results). The black line represents the fluorescence intensity of hEVs incubated with CD73 antibody; this line is superimposable on the red line showing the fluorescence intensity of EVs obtained from lipofectamine-transfected hMSCs (hEVlp). The blue line represents the fluorescence intensity of EVs obtained from CD73-silenced hMSCs (hEVsi). (**C**,**D**) Representative graphs of nanoparticle tracking analysis showing the size distribution of (**C**) hEVlp and (**D**) hEVsi.

MSCs showed spindle-shaped morphology, surface antigen expression, and a capacity for differentiation into osteoblasts and adipocytes in vitro, as previously described [36]. The phenotypes of EVs derived from human MSCs (hMSCs) transfected in the absence (hEVlp) or presence (hEVsi) of a CD73-specific small interfering RNA (siRNA) were evaluated using MACS multiplex bead-based flow cytometry assay (Figure 1A) [37,38]. The cytofluorimetric analyses of hEVlp and hEVsi revealed that the transfection procedure did not modify the phenotype of EVs derived from naïve hMSCs (hEVs, controls). Indeed, both hEVlp and hEVsi were positive for typical MSC markers (CD29, CD44, CD49e, CD105, and CD146) as well as exosomal markers (CD9, CD63, and CD81), but did not express hematopoietic (CD45, CD3, CD8, etc.), endothelial (CD31), or epithelial (CD24 and CD326) markers (Figure 1A). Moreover, cytofluorimetric analyses showed that CD73 expression on hEVsi was downregulated at 72 h after the transfection procedure (Figure 1B).

The mean size of the hEVs was 260 nm, and transfection in the presence or absence of the specific siRNA did not modify the EV size (Figure 1C,D).

**Figure 2:**
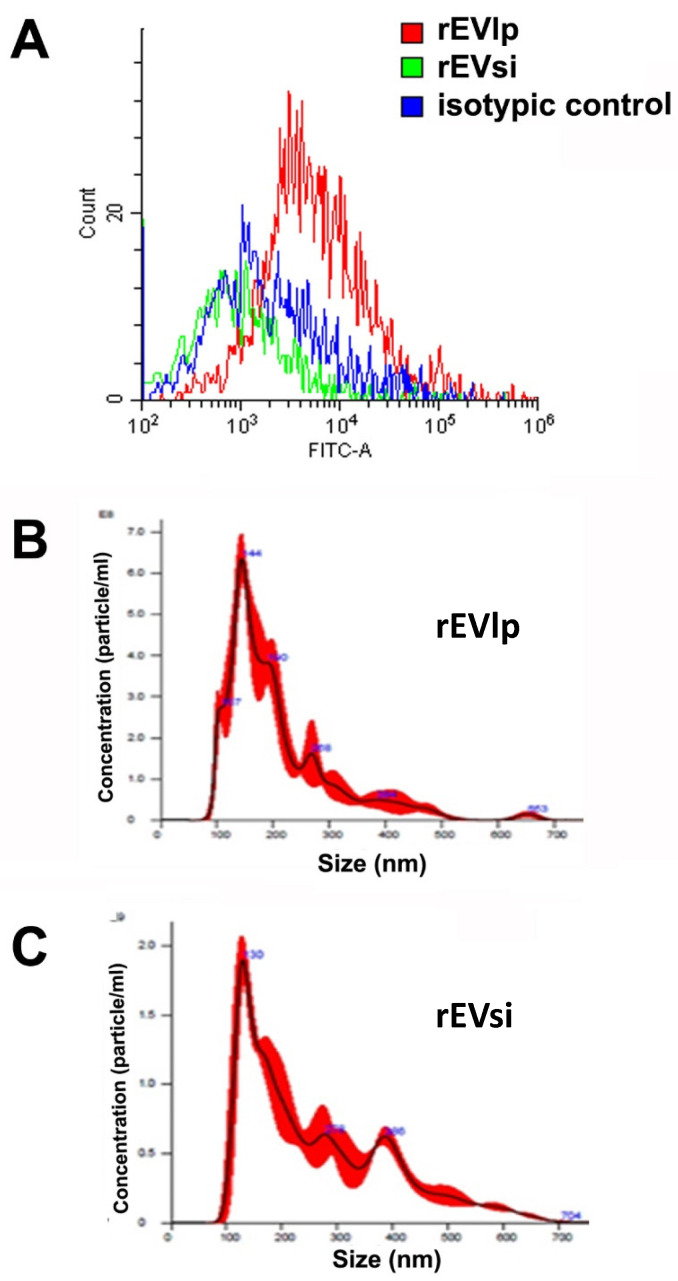
**Characterization of rat extracellular vesicles (EVs).** (**A**) Representative cytofluorimetric analyses of EVs obtained from rat mesenchymal stromal cells (rMSCs) transfected in the presence (rEVsi) or absence (rEVlp) of a CD73-specific small interfering RNA (siRNA) (n = 3 samples analyzed with similar results). The red line represents the fluorescence intensity of rEVlp incubated with an anti-CD73 antibody. The blue line represents the fluorescence intensity of rEVs obtained from CD73-silenced MSCs (rEVsi). The green line is the isotypic control. (**B**,**C**) Representative graphs of nanoparticle tracking analysis showing the size distribution of (**B**) rEVlp and (**C**) rEVsi (n = 3 samples analyzed with similar results).

The downregulation of CD73 was also confirmed on EVs derived from rat MSCs (rMSCs) transfected in the presence of the specific siRNA (rEVsi; Figure 2A). The mean size of the rEVs was 230 nm, and transfection in the presence or absence of the specific siRNA did not modify the EV size (Figure 2B,C).

### 2.2. Cell Proliferation Assay

**Figure 3:**
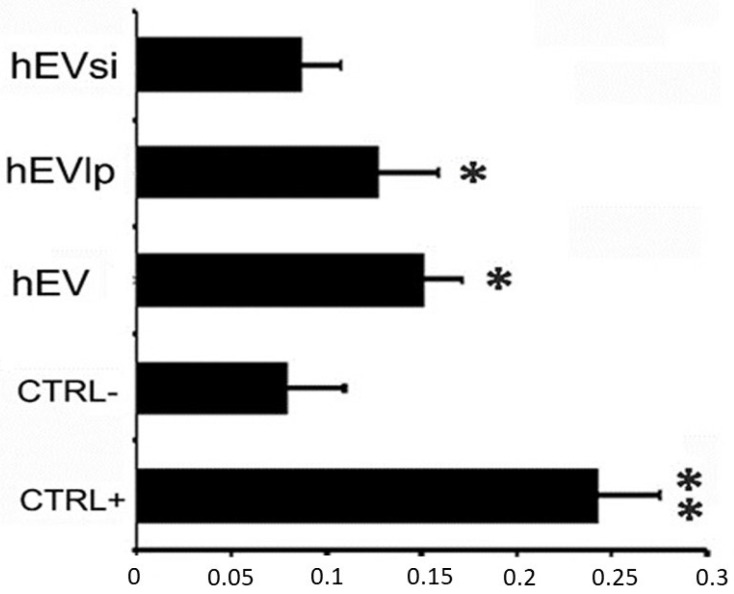
Effects of different types of extracellular vesicles (EVs) on the proliferation of human renal proximal tubular epithelial cells (RPTECs). The effects of the following types of EVs on the proliferation of human RPTECs was assessed using bromodeoxyuridine uptake with respect to the negative control cells (CTRL−, serum-free Dulbecco modified Eagle medium): hEVs, EVs obtained from naïve human mesenchymal stromal cells (hMSCs); hEVlp, EVs obtained from hMSCs transfected in the absence of a CD73-specific small interfering RNA (siRNA); and hEVsi, EVs obtained from hMSCs transfected in the presence of CD73-specific siRNA. Cells cultured in complete medium (renal epithelial basal medium) were used as positive controls (CTRL+). Data are expressed as mean ± SD of the absorbance in 3 different experiments performed in quadruplicate. Analysis of variance with the Newman-Keuls multiple comparison test was performed: ** *p* < 0.001 CTRL+ vs. CTRL− and * *p* < 0.05 hEVs and hEVlp vs. CTRL−.

As shown in Figure 3, human renal proximal tubular epithelial cells (RPTECs) stimulated with hEVs and hEVlp proliferated significantly as compared to the negative control. In contrast, hEVsi did not induce the proliferation of human RPTECs.

### 2.3. EV RNA Profiling

**Figure 4:**
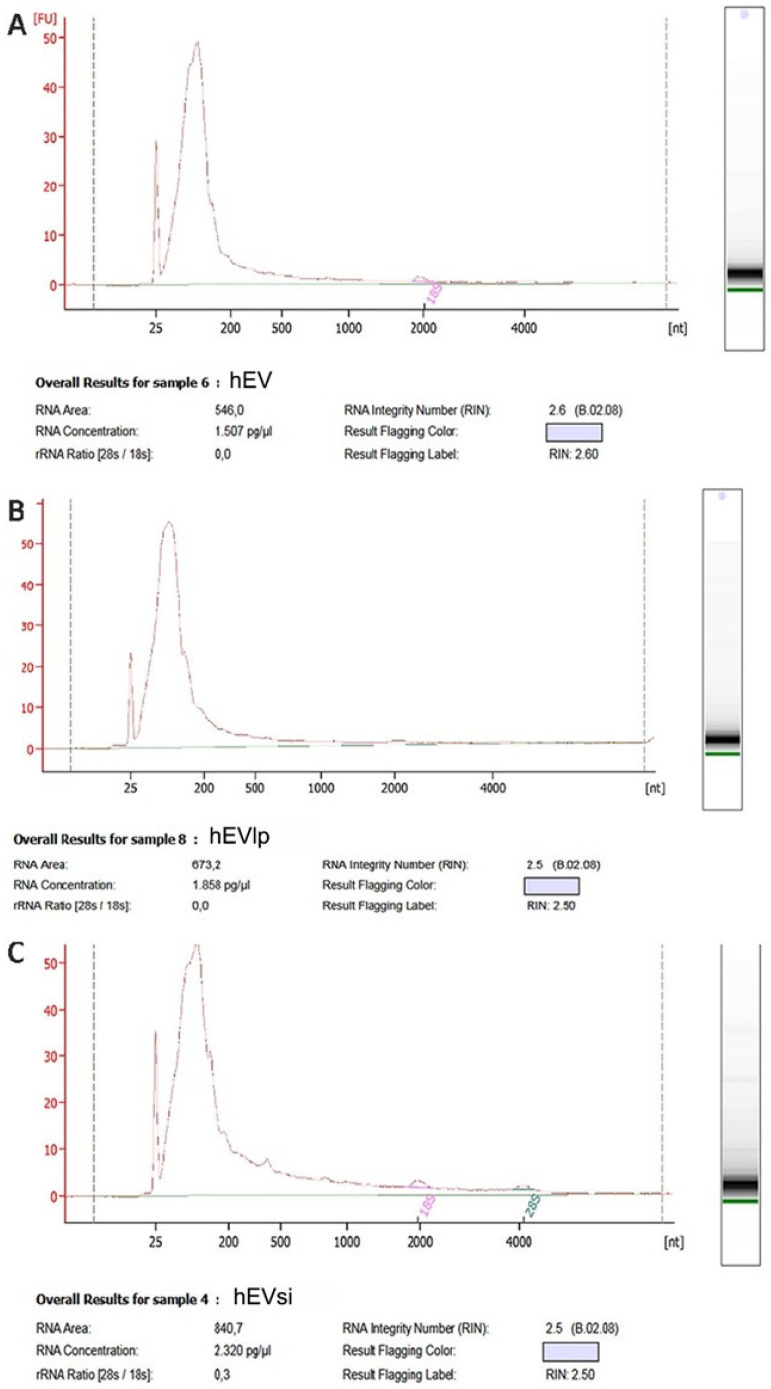
Characterization of RNA content of extracellular vesicles (EVs). Representative bioanalyzer profiles showing the size distribution of the total RNA extracted from (**A**) EVs obtained from naïve human mesenchymal stromal cells (hMSCs), i.e., hEVs, and EVs obtained from hMSCs transfected in the (**B**) absence (hEVlp) or (**C**) presence (hEVsi) of a CD73-specific small interfering RNA (siRNA). N = 3 samples analyzed with similar results. The first peak (left side of each panel) represents an internal standard. The EVs exhibit a relevant peak of small RNAs.

To test the possibility that the transfection procedure affected the RNA content of the EVs, we evaluated the RNA profiles of EVs obtained from naïve hMSCs and hMSCs transfected with or without CD73-specific siRNA. As shown in Figure 4, the integrity of the RNA extracted from the different types of EVs, expressed as the RNA integrity number, did not differ between EVs from naïve and transfected hMSCs. The transfection procedure in the presence or absence of the CD73-specific siRNA did not influence the quantity or quality of the RNA in the EVs.

**Figure 5:**
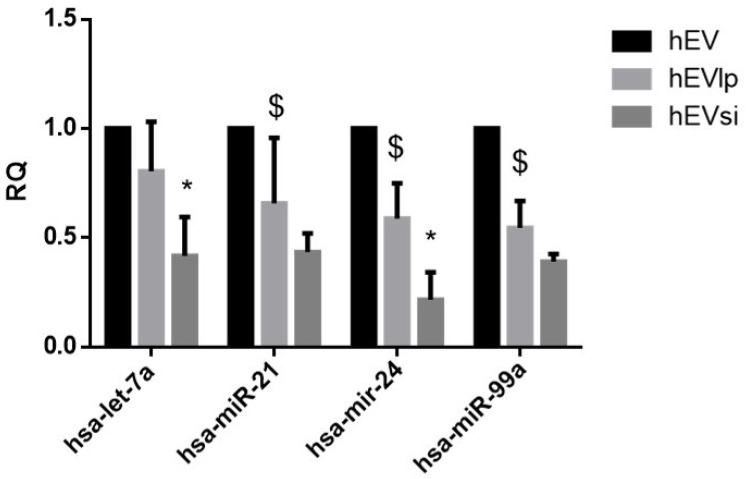
**Real-time polymerase chain reaction (PCR) analyses.** Analyses of the expression levels of selected microRNAs (miRNAs), specifically, hsa-let-7a, hsa-mir-21, hsa-mir-24, and hsa-mir-99a, in extracellular vesicles (EVs) from naïve human mesenchymal stromal cells (hMSCs), denoted as hEVs, and EVs from hMSCs transfected in the absence (hEVlp) or presence (hEVsi) of a CD73-specific small interfering RNA (siRNA). The data are normalized with respect to the expression level of miRNAs in hEVs. * *p* < 0.01 hEVs vs. hEVlp; $ *p* < 0.05 hEVs vs. hEVsi. N = 3 samples analyzed with similar results.

EVs derived from MSCs are enriched in small RNAs [39,40]. The expression levels of some microRNAs (miRNAs) known to be enriched in EVs obtained from bone marrow-derived MSCs were evaluated using real-time polymerase chain reaction (PCR). As shown in Figure 5, the transfection procedure reduced the expression levels of the selected miRNAs. The presence of the CD73-specific siRNA further modified the expression levels of the selected miRNAs.

### 2.4. Tubular Cell Proliferation Index

To evaluate tissue viability, we studied tubular cell proliferation in the perfused kidneys in each group. The tubular cell proliferation index was defined as the ratio between the nuclei expressing PCNA and the total nuclei in each tubule. [36]

**Figure 6:**
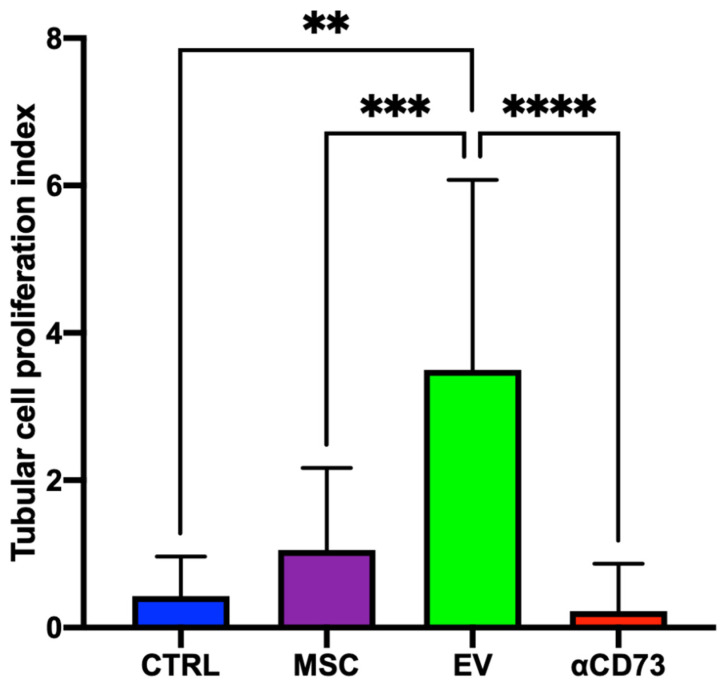
**Tubular cell proliferation index.** The tubular cell proliferation index (IPT) was defined as the ratio between nuclei expressing proliferating cell nuclear antigen and the total nuclei in each tubule in every field analyzed (×40). Data are expressed as mean and standard deviation. ** *p* < 0.01; *** *p* < 0.001; **** *p* < 0.0001. CTRL, control; MSC, mesenchymal stromal cell; EV, extracellular vesicle; αCD73, CD73-silenced group. N = 10 sections from each kidney were analyzed.

After hypothermic perfusion, this index was higher in the EV group (mean ± SD: 3.50 ± 2.57) than in the CTRL (mean ± SD: 0.43 ± 0.53; *p* < 0.01), MSC (mean ± SD: 1.06 ± 1.11; *p* < 0.001), and αCD73 groups (mean ± SD: 0.22 ± 0.65; *p* < 0.0001; Figure 6).

### 2.5. Global Renal Ischemic Damage Score

**Figure 7:**
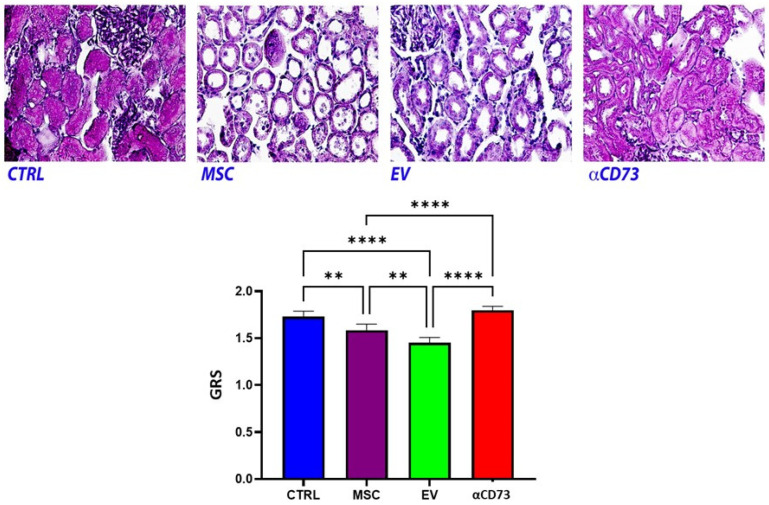
**Global renal ischemic damage score.** Top: Renal morphology. Periodic acid-Schiff staining of representative renal sections from the CTRL, MSC, EV, and αCD73 groups (at ×10 magnification). Bottom: Columns representing the global ischemic damage score (GRS) expressed as mean and 95% confidence interval (** *p* < 0.01; **** *p* < 0.0001). CTRL, control group; MSC, mesenchymal stromal cell group; EV, extracellular vesicle group; αCD73, CD73-silenced group. *N* = 20 sections from each kidney were analyzed.

The global renal score (GRS) was significantly higher in the CTRL group (mean, 1.73; range, 1.67–1.79) than in the MSC group (mean, 1.58; range, 1.52–1.65; *p* < 0.01) or the EV group (mean, 1.45; range, 1.39–1.51; *p* < 0.0001). Furthermore, the EV group showed a significantly lower GRS than the MSC group (*p* < 0.01). As shown in Figure 7 the GRS in the αCD73 group (mean, 1.79; range, 1.75–1.84) was similar to that in the CTRL group, and significantly higher than that in the MSC and EV groups (*p* < 0.0001 for both).

### 2.6. Interrelationship between Tissue and Effluent Purines

**Figure 8:**
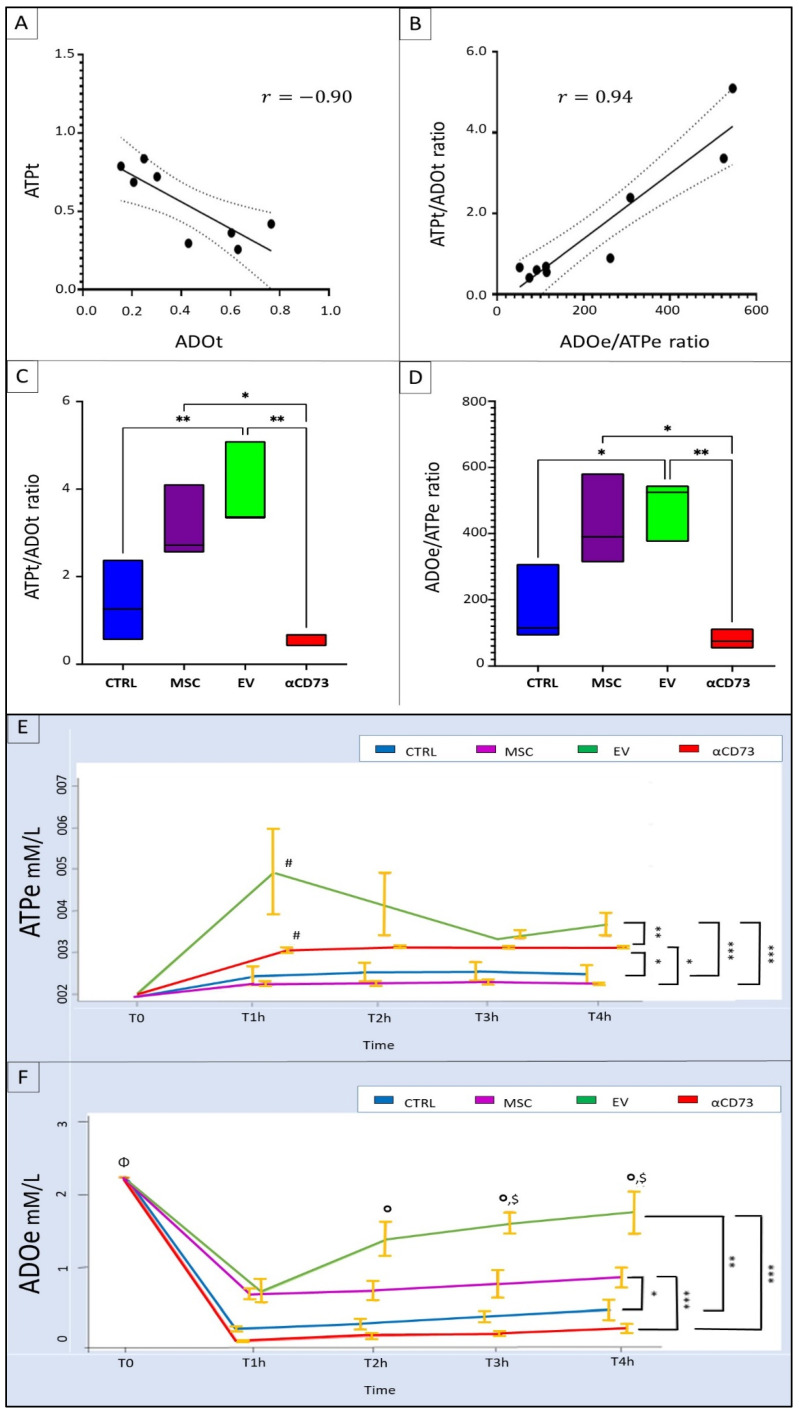
**Tissue and effluent purines.** (**A**) Negative correlation between tissue adenosine (ADO) and adenosine triphosphate (ATP) concentrations (r = −0.90, *p* < 0.001). (**B**) Positive correlation between effluent and tissue purine ratios (r = 0.94, *p* < 0.0001). (**C**) Comparison of the purine ratio in the tissues between different groups. (**D**) Comparison of the purine ratio in the effluent between different groups. (**E**) Variation in effluent ATP levels with time in each group. (**F**) Variation in effluent ADO levels with time in each group. The symbol at the end of the line indicates a significant difference at all time points during hypothermic perfusion. * *p* < 0.05; ** *p* < 0.01; *** *p* < 0.001; #T0 vs. T1h *p* < 0.01; Φ vs. T0 *p* < 0.0001; $ vs. EV T1h *p* < 0.01; ° vs. MSC *p* < 0.001. CTRL, control group; MSC, mesenchymal stromal cell group; EV, extracellular vesicle group; αCD73, CD73-silenced group; T, time since start of hypothermic perfusion (1, 2, 3, or 4 h after the start [T0] of hypothermic perfusion).

We found an inverse correlation between the ATP content and ADO content in the tissues in all groups (r = −0.90; *p* < 0.01) and a direct correlation between the ADO/ATP ratio in the effluent and the ATP/ADO ratio in the tissues in all groups (r = 0.94; *p* < 0.001; Figure 8A,B).

It is important to emphasize that we found a significant positive correlation between the effluent and tissue ATP levels only in the EV group and not in the other groups (r = 1; *p* < 0.0001).

### 2.7. Effluent and Tissue Purine Ratios

At the end of the perfusion time, the ATP/ADO ratio in the tissue and the ADO/ATP ratio in the effluent were higher in the MSC and EV groups than in the CTRL group. These ratios peaked in the EV group (CTRL vs. EV: ATP/ADO ratio in tissue, *p* < 0.01 and ADO/ATP ratio in effluent, *p* < 0.05).

In the αCD73 group, the tissue ATP/ADO ratio and the effluent ADO/ATP ratio were lower than the corresponding ratios in the MSC group (*p* < 0.05 for both) and the EV group (*p* < 0.05 and *p* < 0.01, respectively) and similar to the ratios in the CTRL group (Figure 8C,D).

The ADO and ATP values in the effluents and tissues are reported in Supplementary Table S1.

### 2.8. Time Course of Effluent Purine Levels

We confirmed that the Belzer solution (BS) did not contain ATP at the start of hypothermic perfusion (T0). The effluent ATP level peaked at 1 h after the start of the hypothermic perfusion in the EV group (T0 vs. T1h; *p* < 0.01) and remained higher than the levels in the CTRL and MSC groups at all time points during the perfusion (*p* < 0.001). The effluent ATP levels in the CTRL and MSC groups remained stable and very low during hypothermic perfusion.

In the αCD73 group, the effluent ATP level increased at 1 h after the start of hypothermic perfusion (T0 vs. T1h; *p* < 0.01), but remained stable and lower than the levels in the EV group (*p* < 0.01) and higher than the levels in the CTRL and MSC groups (*p* < 0.05; Figure 8E).

The BS contained ADO at the start of perfusion (T0). The effluent ADO level significantly decreased from the baseline at 1 h after the start of hypothermic perfusion in all groups (T0 vs. T1h; *p* < 0.0001) and remained stable until the end of hypothermic perfusion in the CTRL, MSC, and αCD73 groups. In the EV group, we observed a gradual increase in the effluent ADO level after the first hour, and this level further significantly increased at the third and fourth hours (T1h vs. T3h and T4h; *p* < 0.01). Furthermore, the effluent ADO levels in the EV group were significantly higher than the levels in the CTRL group at all time points during hypothermic perfusion (*p* < 0.01).

In the MSC group, the effluent ADO levels were significantly higher than those in the CTRL group at all time points during hypothermic perfusion (*p* < 0.001) and lower than those in the EV group from the second hour of hypothermic perfusion (*p* < 0.001).

The effluent ADO levels in the αCD73 group were significantly lower than the levels in the MSC and EV groups (*p* < 0.001) and similar to the levels in the CTRL group (Figure 8F).

### 2.9. rMSC Intake of ADO

The ratio of the ADO level at the end vs. the start of incubation (4 h at 4 °C) was lower in BS containing rMSCs than in BS alone (*p* < 0.05; Supplementary Figure S1).

## 3. Discussion

New alternative preservation procedures have been developed to expand the number of transplantable organs and improve their quality [41,42,43,44]. Among all these techniques, hypothermic machine perfusion appears to be an ideal platform for the delivery of different biological and pharmacological agents to organs to be transplanted, and MSCs/EVs may be a promising approach for conditioning kidneys prior to transplantation [12,36,45]. MSCs are a heterogeneous cell population that may be subject to changes in biological functionality after in vitro isolation and expansion. Isolated MSCs from the same source display a heterogeneous transcriptional profile and show a diverse capacity to metabolize extracellular nucleotides, a process which requires the presence of CD73 activity [46,47,48].

CD73 is a classic marker of MSCs and EVs, and it is associated with reparative properties. In fact, ADO derived from the dephosphorylation activity of CD73 can reduce local inflammation and facilitate tissue regeneration [46,49]. The ability of MSCs and EVs to promote tissue regeneration in the setting of acute or chronic renal injury has been widely studied [50,51,52,53,54], but it is not fully understood. Several studies have shown that EVs modify gene expression in target cells by releasing their cargo, which consists of lipids, proteins, and nucleic acids such as miRNAs [55,56].

In this study, we show that CD73 plays an essential role in the regenerative effects of EVs. Our in vitro experiments demonstrated that renal tubular cell proliferation was increased by naïve EVs, but inhibited by CD73-silenced EVs. Furthermore, although we excluded the possibility that the transfection procedure affected the vesicular RNA profile, we found that miRNA expression was reduced in lipofectamine-treated EVs and more significantly reduced in CD73-silenced EVs. Instead, the phenotype and the size of the EVs were not modified by the transfection. 

In a previous study, we discovered the ability of MSCs and EVs delivered via hypothermic machine perfusion to prevent ischemic injury by improving the cell-proliferation rate and reducing tissue damage [12]. Our present ex vivo rat DCD model showed that the hypothermic perfusion of CD73-silenced EVs abrogated the protective effects observed in tissue samples of MSC/EV-treated kidneys.

It is known that the renal damage caused during IRI may be reversible if (i) circulation is restored early; (ii) the cell energy machine is kept running; and (iii) energy metabolites are made available to the cells. In fact, several studies have demonstrated that ATP levels are correlated with less severe ischemic injury of the kidney and liver [57,58]. ATP levels after transplantation are inversely related to warm ischemia time, and low ATP levels are significantly associated with primary graft non-function [59,60]. Hence, current methods of organ preservation aim to contain the energy-depletion rate [59,61].

In our previous experiments, we demonstrated that MSCs/EVs preserve tissue function by providing energy [12,36]. In the present study, we focused on energy metabolism substrates, namely, the purines ATP and ADO. We confirmed that ADO in the tissue was consumed for ATP synthesis in all groups, as shown by the negative correlation between tissue ADO and ATP contents. In the cytosol, ADO can be converted into AMP and subsequently phosphorylated to ADP and ATP; the resulting ATP can be exported out of the cytosol and into the extracellular space [26].

Kidneys conditioned with MSCs and particularly EVs showed the highest tissue availability of ATP, but the levels were high enough to allow the passage of ATP in the effluent only in the EV group, where we found a positive correlation between the ATP levels in the effluent and tissue. In contrast, the absence of CD73 drastically reduced the availability of the energy substrates. The ATP trend in the effluent confirmed the ideal energy status of the EV perfused tissues. Only in EV group, we observed a massive ATP increase in the first hour after perfusion, followed by stabilization at higher levels than in the other groups. The organs conditioned with EVs lacking in CD73 showed significantly reduced ATP levels in the effluent.

It is known that the intracellular ADO level depends on the generation, metabolism, and cellular uptake of ADO. Therefore, intracellular ADO increase is related to extracellular concentrations and oxygen consumption. For this reason, we found understandably, that tissue ATP availability was positively correlated to the effluent ADO levels in all groups.

In our experimental model, the ADO concentrations in the effluent depended both on the ADO present in BS and the ADO generated by the activity of CD73 overexpressed in ischemic renal tissue and (only in the MSC and EV groups) the CD73 expressed on MSC and EV membranes. In all groups, the effluent ADO level decreased dramatically after the first hour of perfusion and remained low until the end of perfusion, except for the EV group. In the EV group, after a slight initial decrease, the ADO level progressively increased until the fourth hour of hypothermic perfusion.

The first-hour drop in all groups may be explained by ADO internalization in the cells and its consumption. The evidence that CD73 plays a pivotal role in the ability of EVs to provide ADO is given by the lowest purine concentration in the effluents of kidneys perfused with EVs missing CD73. In contrast, kidneys perfused with MSCs and especially with EVs had higher effluent levels of ADO than the other groups as a result of the greater CD73 presence.

Other studies have shown the protective effect of ADO produced by CD73 in kidney IRI. In experimental models, the tissue-specific deletion of CD73 and/or the inhibition of its enzymatic activity increases susceptibility to ischemic damage, while the reconstitution of CD73 enzymatic activity and/or the increase of extracellular ADO levels provides protection against IRI, in terms of renal function and tubular damage [23,28,29,30,35,62,63]. There is also evidence that EVs increase ATP and NADH levels via the AMPK and cell energy metabolism pathways [36,64].

Interestingly, in vitro and in vivo studies have shown that the rate of EV incorporation is accelerated in ATP-depleted tubular cells, and MSCs produce EVs enriched in CD39 and CD73 in response to pro-inflammatory cytokines [14,65]. Indeed, the higher ADO and ATP levels found in only the EV-group effluents may be the result of accelerated EV incorporation into cells and subsequent up-regulation of the EV-induced enzymatic energy machine in ischemic tissue [12].

Conversely, the different behavior of the time course of both purines in the effluent of the MSC group could be explained by the metabolic consumption of ADO by MSCs themselves, as shown by our in vitro experiment.

In conclusion, in this study, we identified the mediators of the positive effects of EVs when used as a new strategy for organ preservation. To our knowledge, this is the first report demonstrating the ability of EVs to significantly reduce ischemic damage and improve organ viability by enhancing CD73-adenosinergic signaling. This discovery may be useful for improving organ-preservation techniques and expanding EV-based therapeutic strategies for ischemic diseases.

## 4. Materials and Methods

### 4.1. Cell Cultures, Expansion, Silencing, and Characterization

#### 4.1.1. rMSCs

MSCs were isolated from rat bone marrow flushed from femurs, as previously described [1]. The obtained rMSCs were plated at a density of 16 × 10^4^/cm^2^ in α-minimum essential medium (αMEM) supplemented with 10% murin MesenCult (Voden, Milan, Italy) and 0.1% gentamicin (Sigma-Aldrich, Saint Louis, MO, USA), and maintained at 37 °C in a humidified atmosphere containing 5% CO_2_. After 48 h, non-adherent cells were removed, and the culture medium was replaced twice a week. At confluence, rMSCs were harvested using trypsin-EDTA (Lonza, Copenhagen, Denmark) and propagated at 4000 cells/cm^2^. At passage 4, rMSCs were characterized for morphology and surface antigen expression by using monoclonal antibodies against rat CD11b, CD45, CD73, CD90, CD49e, and CD29 (Becton Dickinson, Franklin Lakes, NJ, USA) and a FACSCalibur flow cytometer (BD Biosciences, San Josè, CA, USA), as previously described [1]. Finally, rMSCs were cryopreserved in phosphate-buffered saline (PBS) supplemented with 25% albumin and 10% dimethyl sulfoxide, at a concentration of 3 × 10^6^ cells/vial.

#### 4.1.2. Human Cells

MSCs from human bone marrow were obtained from Lonza (Basilea, Switzerland) and cultured in MSC Growth Medium (MSCGM) bullet kit (Lonza), according to the manufacturer’s instructions.

Human RPTEC were obtained from Lonza and cultured in Renal Epithelial Cell Basal Medium (REBM, Lonza) supplemented with Renal Epithelial Cell Growth Medium Bullet Kit (REGM, Lonza), according to the manufacturer’s instructions. 

#### 4.1.3. Transient Transfection of rMSCs and hMSCs to Downregulate CD73 Expression

Preliminary set-up experiments were performed using a fluorescent siRNA (Invitrogen, Waltham, MA, USA ) and a commercial kit (RNAiMAX, Invitrogen) to find the optimal transfection conditions for rMSCs and hMSCs. The efficiency of transfection was evaluated using fluorescence-activated cell sorting (FACS) and fluorescence microscopic analyses.

After 24 h of transfection with 5 μL lipofectamine following the manufacturer’s instructions, the highest fluorescent signals were detected using the concentration of 25 nM siRNA for both rMSCs and hMSCs (Supplementary Figure S2).

Using this concentration, we transfected rMSCs (1 × 10^6^/flask) with lipofectamine and a specific siRNA (Invitrogen) to block CD73 expression. We also transfected hMSCs (1 × 10^6^/flask) with lipofectamine with or without CD73 siRNA. CD73 expression was evaluated using FACS analyses at different time points after the transfection procedure. As shown in Supplementary Figure S3A, CD73 expression was downregulated in CD73 siRNA-transfected hMSCs at 24 h after the procedure. After 48 h, CD73 expression was further decreased in both hMSCs and rMSCs transfected with siRNA (Supplementary Figure S3B,C).

Once the conditions that permit CD73 downregulation were established, EVs were collected from transfected and naïve hMSCs and rMSCs, as described below.

### 4.2. EV Isolation and Characterization

**Figure 9:**
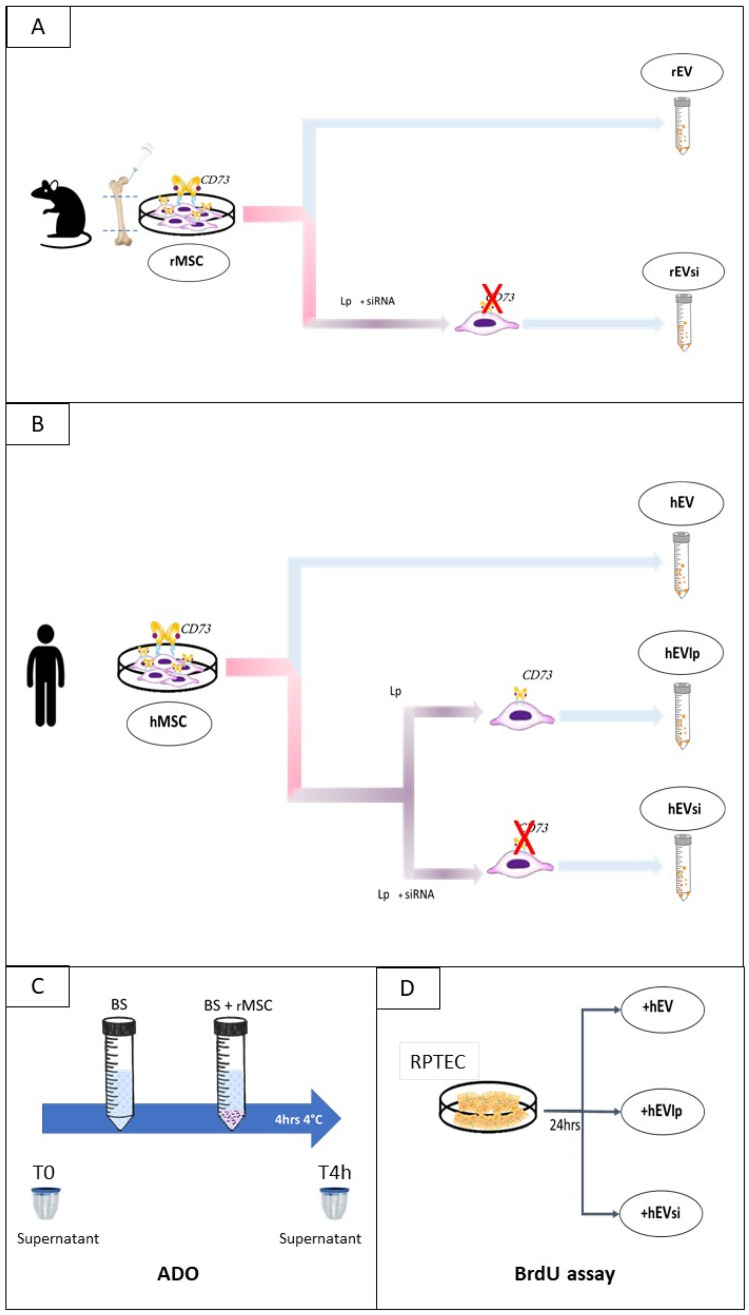
In vitro experimental design. (**A**) Isolation of extracellular vesicles (EVs) from rat mesenchymal stromal cells (rMSCs), naïve cells (rEVs), and CD73-silenced cells (rEVsi). (**B**) Isolation of EVs from human MSCs (hMSCs), naïve hMSCs (hEVs), hMSCs transfected with lipofectamine (lp; hEVlp), and CD73-silenced hMSCs (hEVsi). (**C**) Naïve rMSCs were incubated in Belzer solution (BS) under hypothermic conditions. At the start (T0) and after 4 hours (“h”) (Tend) of incubation, the supernatants of BS and BS + rMSCs were collected to measure the adenosine (ADO) levels. (**D**) Human primary renal proximal tubular epithelial cells (RPTECs) were stimulated with EVs derived from naïve hMSCs (hEVs), lp-transfected hMSCs (hEVlp), and CD73-silenced hMSCs (hEVsi). After 24 h, cell proliferation was evaluated using the bromodeoxyuridine (BrdU) assay.

EVs were obtained by ultracentrifugation, as previously described [12]. In brief, EVs were obtained from the supernatants of rMSCs and hMSCs cultured overnight in Roswell Park Memorial Institute (RPMI) 1640 medium. EVs were isolated from naïve cells (rEVs/hEVs) as well as from lipofectamine-transfected (hEVlp) and CD73-silenced (rEVsi/hEVsi) MSCs at 48 h after the transfection procedure (Figure 9A,B).

Cell debris and apoptotic bodies were removed by centrifugation at 3000× *g* for 20 min followed by microfiltration through a 0.22-mm vacuum filter unit (Millipore, Billerica, MA, USA). The EVs were then purified using ultracentrifugation at 100,000× *g* for 2 h at 4 °C (Beckman Coulter Optima L-100 K, Fullerton, CA, USA). The purified EVs were used fresh, or stored at −80 °C and used after resuspension in RPMI 1640 medium supplemented with 1% dimethyl sulfoxide (Sigma-Aldrich). Analysis of the size distribution and enumeration of the EVs were performed using NanoSight NS300 (NanoSight Ltd., Amesbury, UK) equipped with a 405-nm laser and Nanoparticle Tracking Analysis software (NTA version 3.2) (NanoSight Ltd.).

The particles in the sample were illuminated using a laser light source, and the scattered light was captured by the camera and displayed on the connected computer running NTA software. NTA automatically tracked and sized the particles based on Brownian motion and diffusion coefficient (Dt). Three 30-s videos were recorded to perform the analyses.

EVs were characterized as previously described [7,12]. In brief, the expression of surface markers was evaluated on hEVlp and hEVsi by using a human-specific cytofluorimetric bead-based MACSPlex Exosome Kit (Miltenyi Biotec, Bergisch Gladbach, Germany). The expression of CD73 was checked on rat and human EVs to determine if the transfection procedure reduced this expression. CD73 expression was evaluated using FACS analyses performed with a CytoFLEX flow cytometer (Beckman Coulter, Brea, CA, USA). The instrument was rinsed with particle-free rinse solution for 15 min to eliminate the background, and the analysis was performed using a log scale for forward-scatter and side-scatter parameters. A specific antibody against rat CD73 (Becton Dickinson) was conjugated using an Alexa Fluor Antibody Labeling Kit (Molecular Probes, Life Technology, Carlsbad, CA, USA). For the detection of human CD73, we used a specific antibody directly allophycocyanin (APC)-conjugated (Novus Biologicals, Littleton, CO, USA).

### 4.3. Cell-Proliferation Assay

To determine whether the presence of CD73 on EVs affected the renal cell proliferation rate, we stimulated RPTECs with hEVs, hEVlp, and hEVsi. RPTECs were placed in 96-well plates at a concentration of 3 × 10^3^ cells/plate and starved by incubation with serum-free Dulbecco modified Eagle medium for 6 h. Subsequently, the RPTECs were stimulated with hEVs, hEVlp, and hEVsi at a concentration of 1 × 10^3^/cell for 24 h. Cell proliferation was evaluated using a BrdU Cell Proliferation Assay Kit (Cell Proliferation ELISA, BrdU; Sigma-Aldrich), according to the manufacturer’s instructions (Figure 1D). Three quadrupled experiments were performed for each condition.

### 4.4. EV RNA Profiling

To understand whether the CD73 gene silencing on hMSCs affected the RNA contained in the EVs, we analyzed the miRNA profiles of the transfected EVs. RNA was extracted from hEVlp and hEVsi by using TRIzol LS (Thermo Fisher Scientific, Ltham, MA, USA), according to the manufacturer’s instructions, as previously described [66].

In brief, 250 µL EV suspension was incubated with 750 µL TRIzol LS for 5 min followed by incubation with 250 µL chloroform for 3 min. Subsequently, the samples were centrifuged at 12,000× *g* at 4 °C for 15 min to allow phase separation. The upper aqueous phase was then transferred, and 1.5 times of the total obtained volume of 100% ethanol was added to allow RNA precipitation. MiRNAs were then purified using a miRNeasy Mini Kit (Qiagen, Hilden, Germany), according to the manufacturer’s instructions. RNA concentrations were assessed using a NanoDrop 2000 spectrophotometer (Thermo Fisher Scientific).

The profile of the RNA contained in the EVs was evaluated using the Agilent RNA 6000 Pico Kit and the Agilent 2100 Bioanalyzer instrument (Agilent Technologies, Santa Clara, CA, USA). Quantitative reverse transcription (RT)-PCR was performed as described previously [67]. In brief, RT-PCR was performed using miRCURY LNA Universal RT microRNA PCR (Exiqon-Qiagen, Vedbaek, Denmark), after cDNA synthesis. The miRNA quantification was performed using the miScript Reverse Transcription Kit and miScript SYBR Green PCR Kit (both from Qiagen). The following specific primers for miRNAs known to be particularly enriched in hEVs [39,67] were used:

- hsa-miR-21-5p (TAGCTTATCAGACTGATGTTGA);
- hsa-miR-24-3p (TGGCTCAGTTCAGCAGGAA);
- hsa-let-7a-5p (TGAGGTAGTAGGTTGTATAGTT); and
- hsa-miR-99a-5p (AACCCGTAGATCCGATCTTGTG).

RNU6b (CGCAAGGATGACACGCAA) was used as housekeeping reference gene to normalize the quantitative RT-PCR outputs. The data were normalized with respect to hEVlp, and were represented as relative quantification + SEM.

### 4.5. rMSC Intake of ADO

To verify if during perfusion, MSCs consumed the ADO in BS, we thawed cryopreserved MSCs and re-suspended them in BS at a concentration of 6 × 10^3^ cells/mL. Next, rMSCs were incubated at 4 °C in BS in polypropylene tubes to avoid attachment of the rMSCs to plastic. Samples from 3 tubes of BS with or without rMSCs were collected at the start of the incubation (T0) and after 4 h to assay ADO levels. All the measurements were performed in duplicate (Supplementary Figure S1).

### 4.6. Experimental Design: DCD Kidney Model

Rats were anesthetized using isoflurane 2%–5% (Baxter, Como, Italy). After a midline laparotomy, the left retroperitoneal renal area was exposed, and the lumbar arteries were isolated and sectioned; subsequently, the renal artery and vein were isolated. After 20 min of warm ischemia induced by clamping the renal artery, left nephrectomy was completed with preservation of the renal hilum. The kidneys were then perfused with BS or BS supplemented with rMSCs, rEVs, or rEVsi. DCD kidneys were randomized to the following experimental groups:

CTRL group: n = 4 DCD rat kidneys perfused with BS
MSC group: n = 4 DCD rat kidneys perfused with BS supplemented with rMSCs
EV group: n = 4 DCD rat kidneys perfused with BS supplemented with rEVs
αCD73 group: n = 3 DCD rat kidneys perfused with BS supplemented with rEVsi

**Figure 10:**
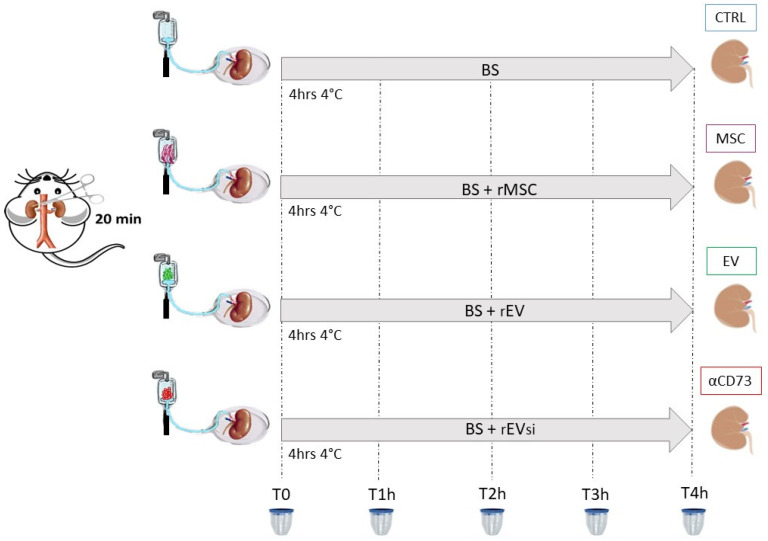
Ex vivo experimental design. Donation after circulatory death was simulated by clamping the rat aorta for 20 min. Then, the kidneys were perfused for 4 hours (“h”) with Belzer solution (BS) at 4 °C (CTRL group) or with BS supplemented with 3 × 10^6^ rat mesenchymal stromal cells (rMSCs; MSC group) or extracellular vesicles (rEVs) isolated from 3 × 10^6^ rMSCs (EV group) or from 3 × 10^6^ CD73-silenced rMSCs (rEVsi; αCD73 group). During the perfusion, the effluents were collected every hour. T0 indicates the start of hypothermic perfusion, and T1h, T2h, T3h, and T4h indicate 1, 2, 3, and 4 h after the start of hypothermic perfusion. The renal tissue was collected at the end of the perfusion.

Continuous hypothermic perfusion was performed for 4 h at 4 °C (Figure 10). Each kidney in the MSC, EV, and αCD73 groups was perfused with 3 × 10^6^ cells or with EVs derived from 3 × 10^6^ rMSCs. The dose was chosen based on our previous experiment [12].

#### 4.6.1. Samples

Effluent fluid was collected and stored at −80 °C at the beginning of the hypothermic perfusion (T0) and at each hour after the start of perfusion (T1h, T2h, T3h, and T4h). At the end of the perfusion (T4h), the kidneys were split into two aliquots; one was fixed in 10% formalin for morphological studies, and the other was stored at −80 °C for biochemical assays. The ATP and ADO levels were measured in the tissue and effluent samples collected.

#### 4.6.2. Histological Studies

##### Tubular Cell Proliferation Index

Paraffin-embedded tissue sections were mounted on poly-l-lysine-coated slides (Dako, Carpinteria, CA, USA), dewaxed in xylol, cleared in a decreasing series of alcohol, and rehydrated with distilled water. Endogenous peroxidase was blocked by treatment with H_2_O_2_ (3.7% vol) followed by H_2_O for 15 min. After 3 washes in 150 mM PBS, the sections underwent microwave antigen retrieval, and were exposed overnight at 4 °C to monoclonal mouse anti-PCNA antibody (1:200, Santa Cruz Biotechnology, Santa Cruz, CA, USA).

After another 3 washes in PBS, the immunocomplex was visualized with biotin-streptavidin-peroxidase complex and 3,3-diaminobenzidine (Dako, Glostrup, Denmark). The sections were lightly counter-stained with Harris hematoxylin. Negative controls were established by omitting the primary antibody and substituting immunoglobulin G for the primary antibodies.

We analyzed 10 non-consecutive sections from each immunostained kidney. The images were captured using a Nikon Eclipse E200 microscope (Amsterdam, The Netherlands) connected to a charge-coupled device (CCD) camera and ImageJ, an image-analysis software (NIH, Bethesda, MD, USA).

The tubular cell proliferation index was defined as the ratio between the nuclei expressing PCNA and the total nuclei in each tubule in every field analyzed (magnification, ×40).

##### Renal Morphology

Twenty subserial cross-sections of each kidney were stained with periodic acid– Schiff and examined by 2 investigators in a double-blind fashion, by using the Nikon Eclipse E200 microscope connected to the CCD camera and Image J software (NIH). GRS was calculated by scoring all tubules observed in at least 10 non-consecutive high-powered fields, as described by Paller et al. [68]. Each lesion was assigned a different score as follows: tubular epithelial flattening, 1 point; brush border loss, 1 point; bleb formation, 2 points; tubular necrosis, 2 points; and tubular lumen obstruction, 2 points. When ≥2 lesions were present in the same tubule, the more severe score was assigned.

#### 4.6.3. Biochemical Assays

##### Tissue Homogenization

Renal tissues were harvested, washed with PBS, and resuspended in 500 μL ATP assay buffer. The tissues were homogenized and centrifuged (4 °C at 13,000× *g*), and the collected supernatants were incubated on ice, for 5 min, with 4 M perchloric acid (PCA) to obtain a final concentration of 1 M PCA in the homogenate solution. The samples were centrifuged again (4 °C at 13,000× *g*), and the excess PCA was precipitated by adding ice-cold 2 M KOH. After the neutralization, the pH was adjusted to 6.5–8 when required, and after further centrifugation (4 °C at 13,000× *g* for 15 min), the tissue supernatants were collected and stored at −80 °C.

##### Separation of ADO

Chromatographic analyses of biological samples were performed with a Jasco PU 980 HPLC gradient system (Japan Spectroscopic, Tokyo, Japan), by using a C18 (Waters, Etten-Leur, The Netherlands) reversed-phase column (150 × 4.6 mm I.D.; 3.5 µm particle size) equipped with a C18 (4.0 × 30 mm I.D.) wide-pore cartridge (Phenomenex, Torrance, CA, USA). Solvent A consisted of double-distilled water (Millipore, Bedford, MA, USA) containing 0.05% tetrabutylammonium (TBA; Sigma-Aldrich), and solvent B consisted of acetonitrile (Sigma-Aldrich) containing 0.05% TBA.Separations were carried out by mixing solvents A and B to form a linear gradient from 0% to 100% B in 35 min. The flow rate was 0.8 mL/min. At the end of each run, the column was washed for 2 min with 100% solvent B and then re-equilibrated with solvent A for 5 min.

Unless otherwise stated, a 20-µL sample volume (of effluent or homogenized tissue) was injected into the column for each run. The analyses were monitored using the UV absorption at 260 nm, and the amount of analytes was determined from their peak areas and converted to molar quantities by reference to a calibration curve generated by injecting known, scalar amounts of standard ADO (Sigma-Aldrich).

##### ATP Measurement

To avoid interferences in the enzyme-linked immunosorbent assay, supernatants of renal tissues were deproteinised according to the manufacturer’s instructions (ab83355, ATP Assay Kit; Abcam, Cambridge, UK). Both the supernatants and the effluents were incubated with the ATP probe. Absorbance was detected at 580 nm by using a microplate reader (Sunrise Tecan Trading AG, Lifescience, Switzerland). The results were expressed as millimoles per liter (mM).

### 4.7. Statistical Analysis

Continuous variables were described as mean and range, standard deviation, or standard error if they were normally distributed and as median and interquartile range (p25–p75) if their distribution was skewed. Categorical variables were expressed as counts and percentages. Correlations between continuous variables were studied using the Spearman rho (ρ) coefficient and represented with scatterplots. Parametric variables were compared between 2 groups by using the Student unpaired *t*-test. Comparisons of continuous variables between more than 2 groups were performed using analysis of variance or the Kruskal-Wallis test with the Tukey, Dunn, or Newman-Keuls multiple comparison test. Appropriate linear regression models for repeated data over time were used to evaluate the between-group and within-group comparisons over time for the outcome variables under study. Adequate graphical representation of data changes over time was accomplished using time-series plots.

Statistical significance was set at alpha = 0.05 (statistical significance was reported for *p* values < 0.05). All tests were two-sided. Analyses were performed using STATA statistical software version 13 or later (STATA Corp., College Station, TX, USA).

## Data Availability

Not applicable.

## Supplementary Materials

The following supporting information can be downloaded at https://www.mdpi.com/article/10.3390/ijms231810681/s1.
