# Supplementary material for: CD73-Adenosinergic Axis Mediates the Protective Effect of Extracellular Vesicles Derived from Mesenchymal Stromal Cells on Ischemic Renal Damage in a Rat Model of Donation after Circulatory Death
Source: Int J Mol Sci. 2022 Sep 14;23(18):10681. doi: 10.3390/ijms231810681 (PMC9501320; doi:10.3390/ijms231810681)
Supplement: Supplementary file 1 [file ijms-23-10681-s001.zip › ijms-1886703-supplementary.pdf]

## Supplementary Materials Grignano et al.

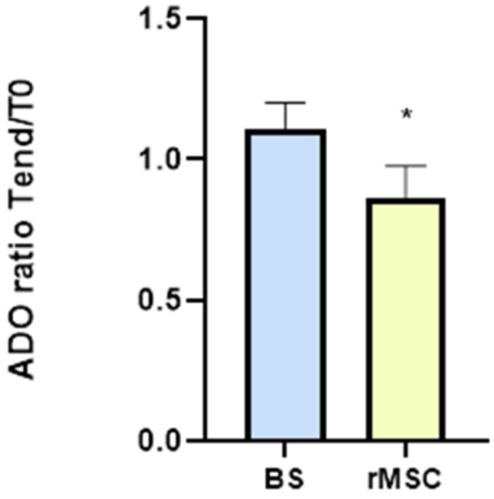


**Supplementary Figure S1. Adenosine (ADO) intake by rat mesenchymal stromal cells (rMSCs).** Supernatant ADO levels in Belzer solution (BS) alone and in BS containing rMSCs at the beginning (T0) and after 4 h (Tend) of incubation at 4 °C. The results are expressed as the Tend/T0 ratio (mean ± SD) (* $p < 0.05$) in 3 different experiments performed in duplicate.

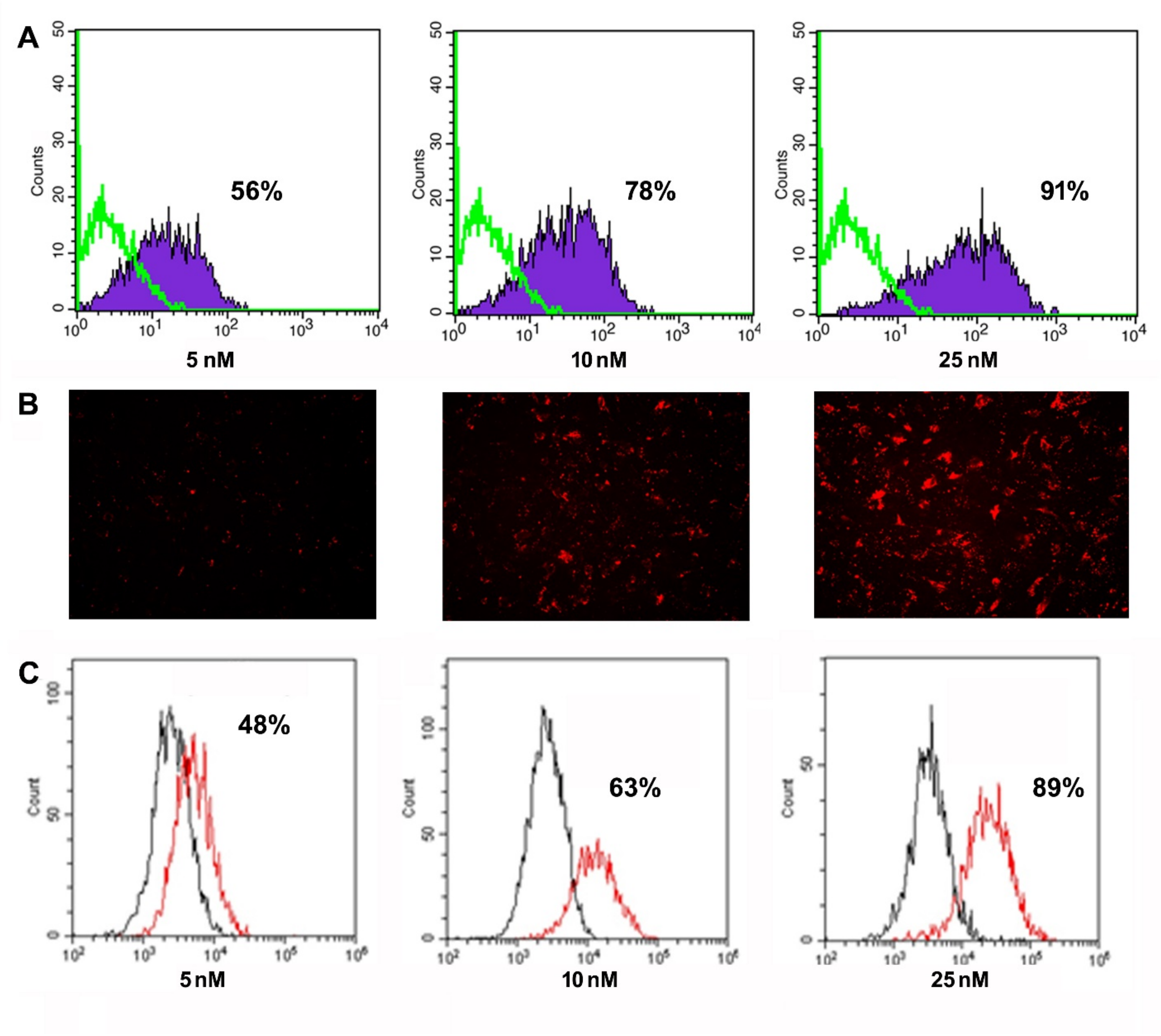


**Supplementary Figure S2.** Representative images of red fluorescence expression in rat mesenchymal stromal cells at 24 h after transfection with lipofectamine in the presence of different concentrations of fluorescent control small interfering RNA (siRNA; 5, 10, and 25 nM), as evaluated using cytofluorimetric analysis (**A**) and fluorescent microscopy (**B**). (**C**) Red fluorescence expression in human mesenchymal stromal cells at 24 h after transfection with lipofectamine and different amounts of control red siRNA (5, 10, and 25 nM), as evaluated using cytofluorimetric analysis. Three experiments with similar results have been performed.

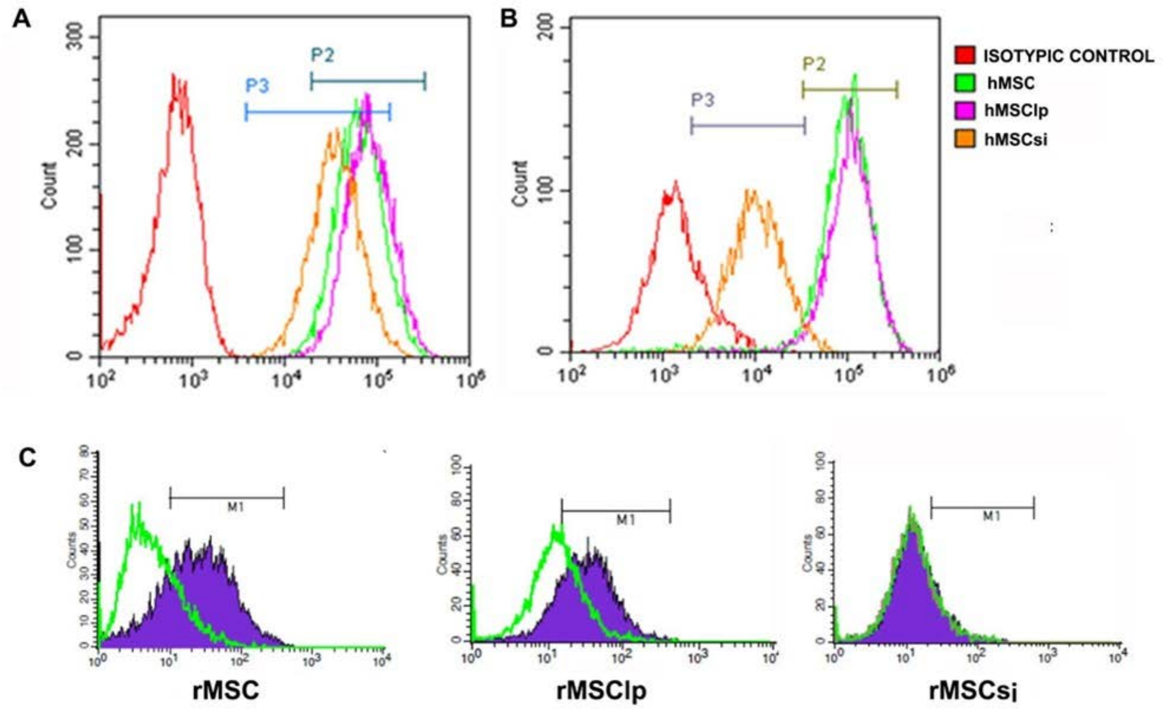


**Supplementay Figure S3.** CD73 expression on mesenchymal stromal cells (MSCs) after the transfection procedure. Representative cytofluorimetric analyses of CD73 expression on human MSCs (hMSCs) at 24 (A) and 48 h (B) after transfection. The red line represents the fluorescence intensity of hMSCs not exposed to the specific CD73 antibody (isotypic control). The green line represents the fluorescence intensity of hMSCs incubated with CD73 antibody, and is superimposable on the purple line that shows the fluorescence intensity of hMSCs transfected with only lipofectamine (hMSClp). The orange line represents the fluorescence intensity of CD73-silenced MSCs (hMSCsi). (C) Representative cytofluorimetric analyses of CD73 expression on control rat MSCs (rMSCs) and rMSCs transfected in the presence or absence of specific siRNA (rMSCsi and rMSClp, respectively) at 48 h after the procedure. The green lines represent the fluorescente intensity of istotypic controls. The purple diagrams rapresent the fluorescent intensity of rMSCs, rMSClp and rMSCsi, incubated with CD73 antibody. Three experiments with similar results have been performed in duplicate.

| Time Point | Group | $ADO_{effluent}$ mean ± SD | $ATP_{effluent}$ mean ± SD | $ADO_{effluent}/ATP_{effluent}$ median (IQR) | $ATP_{tissue}/ADO_{tissue}$ median (IQR) |
|---|---|---|---|---|---|
| T0 | All | 2.33 ± 0.03 | Undetected | | |
| T1h | CTRL | 0.15 ± 0.50 | $2.32 \times 10^{-3} \pm 3.77 \times 10^{-4}$ | | |
| | MSC | 0.60 ± 0.16 | $2.20 \times 10^{-3} \pm 1.40 \times 10^{-4}$ | | |
| | EV | 0.67 ± 0.28 | $4.84 \times 10^{-3} \pm 1.14 \times 10^{-3}$ | | |
| | αCD73 | 0.08 ± 0.02 | $2.99 \times 10^{-3} \pm 6.98 \times 10^{-5}$ | | |
| T2h | CTRL | 0.22 ± 0.12 | $2.40 \times 10^{-3} \pm 4.08 \times 10^{-4}$ | | |
| | MSC | 0.68 ± 0.23 | $2.18 \times 10^{-3} \pm 9.42 \times 10^{-5}$ | | |
| | EV | 1.39 ± 0.40 | $4.02 \times 10^{-3} \pm 8.80 \times 10^{-4}$ | | |
| | αCD73 | 0.15 ± 0.06 | $3.05 \times 10^{-3} \pm 1.23 \times 10^{-5}$ | | |
| T3h | CTRL | 0.32 ± 0.13 | $2.48 \times 10^{-3} \pm 4.26 \times 10^{-4}$ | | |
| | MSC | 0.77 ± 0.37 | $2.23 \times 10^{-3} \pm 5.24 \times 10^{-5}$ | | |
| | EV | 1.61 ± 0.26 | $3.24 \times 10^{-3} \pm 4.02 \times 10^{-4}$ | | |
| | αCD73 | 0.18 ± 0.04 | $3.04 \times 10^{-3} \pm 5.11 \times 10^{-5}$ | | |
| T4h | CTRL | 0.41 ± 0.24 | $2.36 \times 10^{-3} \pm 4.09 \times 10^{-4}$ | 114.50 (91.61–308.50) | 1.26 (0.06–2.27) |
| | MSC | 0.86 ± 0.27 | $2.18 \times 10^{-3} \pm 4.03 \times 10^{-5}$ | 390.30 (312.80–582.50) | 2.72 (2.58–3.78) |
| | EV | 1.75 ± 0.51 | $3.58 \times 10^{-3} \pm 4.93 \times 10^{-4}$ | 524.80 (374.90–545.70) | 3.36 (3.33–5.10) |
| | αCD73 | 0.24 ± 0.09 | $3.06 \times 10^{-3} \pm 1.42 \times 10^{-5}$ | 74.69 (52.19–113.20) | 0.67 (0.41–0.69) |

**Supplementary Table S1.** ADO and ATP values in effluents and tissues.

ADO, adenosine; ATP, adenosine triphosphate; SD, standard deviation; IQR, interquartile range; CTRL, control group; MSC, mesenchymal stromal cell group; EV, extracellular vesicle group; αCD73, CD73-silenced group; T, time since start of hypothermic perfusion (1, 2, 3, or 4 h after the start [T0] of hypothermic perfusion).
